# Syndromes drépanocytaires majeurs et infections associées chez l ’enfant au Burkina Faso

**DOI:** 10.11604/pamj.2017.26.7.9971

**Published:** 2017-01-04

**Authors:** Sonia Douamba, Kisito Nagalo, Laure Tamini, Ismaël Traoré, Madibèlè Kam, Fla Kouéta, Diarra Yé

**Affiliations:** 1 Centre Hospitalier Universitaire Pédiatrique Charles de Gaulle, Ouagadougou, Burkina Faso; 2 Unité de Formation et de Recherche en Sciences de la Santé, Université de Ouagadougou, Ouagadougou, Burkina Faso; 3 Ministère de la Santé, Ouagadougou, Burkina Faso

**Keywords:** Syndrome drépanocytaire majeur, infections, enfant, Ouagadougou, Major sickle cell syndrome, infections, child, Ouagadougou

## Abstract

**Introduction:**

Le but de cette étude était d’étudier les infections chez les enfants présentant un syndrome drépanocytaire majeur.

**Méthodes:**

Étude hospitalière monocentrique, rétrospective descriptive sur dix années menée à Ouagadougou, Burkina Faso. Étaient inclus tous les enfants porteurs d'un syndrome drépanocytaire majeur (homozygote SS et double hétérozygote SC, SD_Punjab_, Sβ thalassémique, SO_Arab_ et SE) hospitalisés pour une infection bactérienne confirmée à la microbiologie.

**Résultats:**

Cent trente trois patients répondaient à nos critères d’inclusion. Le phénotype SS représentait 63,2% des cas et le SC 36,8%. La fréquence des infections était de 21,8%. Celles-ci touchaient dans 45,9% des cas les enfants âgés de 0 à 5 ans. Les signes les plus fréquents étaient les douleurs ostéoarticulaires (42,1%), la toux (25,7%), les douleurs abdominales (23,3%), la pâleur (43,6%). Les broncho-pneumopathies (31,6%), le paludisme (16,5%), les ostéomyélites (12,8%) et les septicémies (10,5%) étaient les principaux diagnostics trouvés. Les agents pathogènes isolés étaient *Streptococcus pneumoniae* (35,5%) et *Salmonella sp* (33,3%). Les céphalosporines de 3e génération étaient les antibiotiques les plus fréquemment prescrits. Le taux brut de mortalité était de 7,5%.

**Conclusion:**

Les infections bactériennes et le paludisme dominent le tableau des infections chez l'enfant drépanocytaire majeur au Centre Hospitalier Universitaire Pédiatrique Charles De Gaulle. Les auteurs recommandent la mise en place d’un programme national de prise en charge de la drépanocytose, ce qui permettrait de prévenir voire réduire la survenue des infections chez les enfants drépanocytaires.

## Abstract (en)

**Introduction:**

This study aims to investigate infections in children with major sickle cell syndrome.

**Methods:**

We conducted a monocentric descriptive retrospective hospital study in Ouagadougou, Burkina Faso, over a ten-year period. All children with major sickle cell syndrome (homozygous SS and double heterozygous SC, SD_Punjab_, Sβ thalassemic, SO_Arab_ and SE) hospitalized for microbiologically confirmed infections were enrolled in the study.

**Results:**

One hundred and thirty-three patients met our inclusion criteria. The SS phenotype accounted for 63.2% of cases and SC 36.8%. The frequency of infections was 21.8%. In 45.9% of cases, these affected children aged 0-5 years. The most frequent signs were osteoarticular pain (42.1%), cough (25.7%), abdominal pain (23.3%), pallor (43.6%). The major diagnoses were bronchopneumonia (31.6%), malaria (16.5%), osteomyelitis (12.8%) and septicemia (10.5%). The isolated pathogenic organisms were Streptococcus pneumoniae (35.5%) and Salmonella spp (33.3%). Third generation cephalosporins were the most commonly prescribed antibiotics. Gros mortality rate was 7.5%.

**Conclusion:**

Bacterial infections and malaria dominate the clinical picture of infections in children with major sickle cell syndrome at the at the Pediatrics University Hospital Center Charles De-Gaulle. This study highlights the importance of establishing a national program for the management of sickle-cell anemia, which could help prevent or reduce the occurrence of infections in children with sickle cell syndrome.

## Introduction

La drépanocytose est une maladie génétique cosmopolite dont la transmission se fait selon le mode de l’hérédité autosomique récessive. Elle est due à une anomalie de structure de l'hémoglobine qui conduit à la formation de l'hémoglobine S anormale (Hb S). Les syndromes drépanocytaires majeurs sont représentés par les homozygotes SS et doubles hétérozygotes SC, SD_Punjab_, Sß thalassémique, SO_Arab_ et SE. Les infections qui émaillent de manière fréquente l’évolution de la maladie mettent en jeu le pronostic vital des enfants qui présentent un syndrome drépanocytaire majeur dans nos pays à ressources limitées. Au Burkina Faso, la prévalence de l’anomalie dans la population générale serait comprise entre 5 et 10% et la prise en charge des enfants affectés n’est pas encore bien organisée. Une meilleure connaissance des infections rencontrées chez ces enfants dans notre contexte devrait permettre d'améliorer cette prise en charge et d'envisager des mesures de prévention plus appropriées.

## Méthodes

**Cadre de l'étude:** L’étude s’est déroulée dans les services de pédiatrie médicale et de chirurgie pédiatrique du Centre Hospitalier Universitaire Pédiatrique Charles De Gaulle (CHUP-CDG) de Ouagadougou au Burkina Faso.

**Méthode:** Il s’agissait d’une étude monocentrique, rétrospective et descriptive sur une période de dix ans allant de Janvier 2002 à Décembre 2012.

**Critères d'inclusion:** Ont été retenus pour notre étude, tous les enfants présentant un syndrome drépanocytaire majeur SS ou SC, hospitalisés dans une unité d'hospitalisation avec à l’admission une fièvre (température corporelle ≥ 38,5° C), des signes infectieux (toux, diarrhée, douleurs abdominales, brulures mictionnelles etc.…) chez qui des examens complémentaires à visée infectieuse étaient réalisés (hémogramme, goutte épaisse, hémoculture, coproculture, examen bactériologique du LCR, des urines, de liquide pathologique, CRP, radiographie du thorax).

**Critères de non inclusion:** Les dossiers avec un taux de complétude des variables étudiées inférieur à 70% n'ont pas été retenus, ainsi que ceux des patients drépanocytaires admis pour d’autres complications évolutives.

**Recueil et traitement des données:** Les données étaient recueillies sur une fiche individuelle à partir des dossiers cliniques et des registres d'hospitalisation. Les principales variables étudiées étaient: l'âge, le sexe, le type d'hémoglobine, le délai de consultation, la provenance des patients, le revenu socio-économique des parents, les antécédents vaccinaux, le motif de consultation, les signes cliniques, le type de germe isolé, le diagnostic retenu, le type de traitement, et le mode évolutif.

**Définitions opérationnelles:** Le niveau socio-économique des parents (NSE) était classé en : bas (élèves, étudiants, employés de maison, artisans, sans-emploi, parents aux revenus irréguliers, cultivateurs), moyen (cadres moyens du privé et du public, commerçants, militaires et paramilitaires), et élevé (cadres supérieurs, opérateurs économiques).

**Analyse des données:** Les données étaient saisies et analysées à l'aide du logiciel de traitement SPHINX LEXICA version 5. Les comparaisons étaient faites avec les tests du Chi-deux et de Fischer au seuil de signification de 5% (les différences étaient considérées statistiquement significatives pour p< 0,05).

## Résultats

### Données épidémiologiques

Nous avons colligé 611 dossiers d'enfants porteurs d'un syndrome drépanocytaire majeur hospitalisés au CHUP-CDG durant la période d'étude et 133 répondaient aux critères d’inclusion, soit une fréquence hospitalière de 21,8% d’infections. L’âge moyen des enfants était de 6,7 ± 2,9 ans, avec des extrêmes de 10 mois et 15 ans. La tranche d’âge de 0-5 ans représentait 47,4% (n = 63) de l’échantillon et les patients âgés de plus de 5 ans constituaient 52,6% (n = 70). Il y avait 69 patients de sexe féminin (51,9%) et 64 de sexe masculin (48,1%) soit un sex-ratio de 0,92. La distribution selon le profil phénotypique de l'hémoglobine montrait 63,2% de patients SS (n = 84) et 36,8% de patients SC (n = 49). L’épisode infectieux a été la circonstance de découverte de la drépanocytose chez 24,8% des patients (n = 33) soit 27,4% de patients SS versus 20,4% de patients SC. Sur 100 patients qui se savaient drépanocytaires avant la présente hospitalisation, 53% avaient un suivi médical régulier, 36% n’étaient pas suivis régulièrement, 11% étaient indéterminés. Si on considère le calendrier du Programme Élargi de Vaccination (PEV) 2012 au Burkina Faso, 81,2% des patients étaient à jour.de leurs vaccins. Le vaccin antipneumococcique recommandé par l’OMS chez les drépanocytaires ne faisait pas partie du PEV mais il était administré à 44,5% des patients sur prescription médicale (Tableau 1).

**Tableau 1:** Répartition selon les principaux vaccins hors-PEV reçus par les patients

Vaccin reçu	Effectif	Fréquence (%)
Anti pneumococcique	16	44,5
Anti méningococcique	8	22,2
Anti typhique	7	19,5
Anti *Haemophilus influenzae b*	3	8,3
Anti hépatite virale B	2	5,5
Total	36	100

### Données cliniques

*Délai de consultation:* le délai moyen de consultation était de 10 ± 3,4 jours, avec un délai minimum de 0 jour et un maximum de 90 jours. Le délai moyen de consultation était significativement plus long chez les enfants dont les parents avaient un niveau socio-économique (NSE) bas (12,5 et 9,5 jours respectivement pour le père et la mère) comparé à celui des enfants à NSE élevé (p = 0,02).

*Motifs de consultation:* la répartition selon la survenue des principaux motifs de consultation chez les 133 patients objectivait 42,9% de douleurs ostéoarticulaires suivies de la toux avec 25% et des douleurs abdominales dans 23,3% des cas. La fièvre était associée aux différents motifs de consultation dans 95,5% des cas. Les mêmes motifs de consultation étaient retrouvés pour les deux phénotypes d’hémoglobine SS et SC. Une analyse des facteurs associés aux motifs de consultation et à l’âge des patients montrait 31,7% de toux chez les 0 à 5 ans et 55,7% de douleurs ostéoarticulaires chez ceux qui étaient âgés de plus de 5 ans. ; La différence constatée était statistiquement significative (p = 0,012).

*Examen clinique:* l’état général était bon chez 79,7% des patients à leur admission. La pâleur conjonctivale était présente chez 43,6% des patients à l’entrée‚ l'ictère était présent chez 12%. La température moyenne était de 39,3 ± 0,7 °C avec des extrêmes de 38,5 et 41,5 °C. Une splénomégalie (42,1%), une hépatomégalie (30,1%) et des râles à l'auscultation pulmonaire (28,6%) étaient retrouvés à l’examen physique des patients. Si la splénomégalie, l’hépatomégalie et les râles à l’auscultation pulmonaire étaient plus fréquents chez les patients drépanocytaires SS, une tuméfaction ostéoarticulaire était plus fréquente chez les drépanocytaires SC mais la différence observée n'était pas statistiquement significative (p = 0,675).

### Données d’examens complémentaires

*Hémogramme:* une hyperleucocytose était retrouvée chez 84,9% des patients avec un taux moyen de globules blancs de 22945 ± 3190 /mm^3^ avec des extrêmes allant de 6400 à 78600/mm^3^. Le taux moyen d’hémoglobine était de 6,7 ± 1,3 g/dl avec un minimum de 2,5 g/dl et un maximum de 10 g/dl.

*Examens biologiques:* la goutte épaisse réalisée chez tous les 133 patients et était positive dans 12,8% des cas. L’hémoculture était réalisée chez 37,6% des patients (n = 50) avec un résultat positif de 38%. L'uroculture était réalisée dans 19,5% des cas (n = 26) avec une positivité de 23,1%. La coproculture était réalisée dans 13,5% cas (n = 28) avec un taux de positivité de 75%. L’examen parasitologique des selles était réalisé par 16,5% (n = 22) des patients avec un taux de positivité de 77,3%. À partir de 50 hémocultures et d’un prélèvement de liquide ostéoarticulaire, les bactéries isolées étaient *Streptococcus pneumoniae* (35,3%) et *Salmonella sp*(33,3%). Ces deux pathogènes étaient retrouvés à tous les âges, sans différence significative. Dans les selles de 17 patients, l'examen parasitologique a permis d'isoler *Giardia intestinalis* dans huit cas et *Entamœba histolytica* dans quatre cas.

*Examens d'imagerie :* la radiographie pulmonaire était réalisée chez 51,6% des patients et montrait des images d’anomalies dans 76,5% des cas. L'échographie abdominale était réalisée chez 27,4% des patients avec 73,5% d’anomalies. La radiographie ostéoarticulaire était réalisée chez 18,4% des patients et elle était anormale dans 86,9% des cas.

### Données diagnostiques

*Les principales infections:* le Tableau 2 indique la fréquence des infections chez les enfants présentant un syndrome drépanocytaire majeur. Les principales infections diagnostiquées étaient par ordre de fréquence les broncho-pneumopathies (31,6%), le paludisme (16,5%), les ostéomyélites (12,8%) et les septicémies (10,5%).

**Tableau 2:** Fréquence des infections associées aux syndromes drépanocytaires majeurs hospitalisés à Ouagadougou, Burkina Faso

Diagnostic	Effectif	Fréquence (%)
Bronchopneumopathie	42	31,6
Paludisme	22	16,5
Ostéomyélite^+^	17	12,8
Septicémie	14	10,5
Parasitose intestinale	13	9,8
Méningite	10	7,5
Fièvre typhoide	9	6,8
Infection urinaire	5	3,8
Rhinopharyngite	4	3,0
Cholécystite	3	2,3
Ostéoarthrite	3	2,3
Rhino-bronchite	2	1,5
Otite	2	1,5
Hépatite	2	1,5
Abcès hépatique	1	0,8
Varicelle	1	0,8

*Répartition des diagnostics selon l’âge de l’enfant:* les broncho-pneumopathies étaient le premier diagnostic dans toutes les tranches d’âges. Elles étaient suivies par les septicémies chez les patients de moins de 5 ans et du paludisme pour les patients âgés de 5 ans et plus. Il y avait des cas de paludisme dans toutes les tranches d’âges‚ sans toutefois noter des formes neurologiques. Les patients de plus de 5 ans ont présenté 76,5% des ostéomyélites versus 23,5% pour les moins de 5 ans avec une différence statistiquement significative (p = 0,05). Sur 14 cas de septicémie, 71,4% concernaient les patients de la tranche d’âge de 0-5 ans et les bactéries identifiées étaient le pneumocoque (42,9%) et la salmonelle (28,5%). Nous avons noté 12% (n = 16) de cas de co-infection dont la plus fréquente est l’association du paludisme avec d’autres infections bactériennes. Sur l’ensemble de nos patients‚ 96 ont présenté en plus des infections‚ une autre complication aigue dans 72,2% des cas. La crise vaso-occlusive était la complication aigue associée à l’infection dans 72,9% des cas.

### Données thérapeutiques

*Antibiothérapie:* une antibiotherapie probabiliste était prescrite chez 96,2% (n = 128) des patients. Les prescriptions étaient adaptées aux résultats biologiques dans 38,3% (n = 49) des cas. La ceftriaxone (64,6%), la gentamicine (48,9%), l’association amoxicilline-acide clavulanique (16%) étaient les antibiotiques les plus prescrits. Les antibiotiques étaient associés dans 95 cas (71,4%). Les patients à goutte épaisse positive ont bénéficié d’un traitement comprenant l’artésunate en forme injectable ou la quinine injectable en fonction de la présence d’une anémie. Le traitement chirurgical a concerné 11,3% des patients (n = 15).

### Données évolutives

*Durée d’hospitalisation:* la durée moyenne d'hospitalisation des patients était de 8,3 ± 1,8 jours avec des extrêmes de 2 et 90 jours. Cette durée était supérieure à 8 jours chez 56,4% des enfants.

*Sortie d’hospitalisation:* le mode de sortie était normal pour 86,5% des cas et nous avons noté 7,5% de taux de mortalité. Parmi les décès, 80% étaient du phénotype SS et 20% du phénotype SC. Les décès concernaient 70% des patients de 0-5 ans versus 30% ceux de 6-10 ans; dans 60% (n = 6), il s’agissait de filles et 40% (n = 4) de garçons. Les patients chez qui l’évolution était favorable consultaient dans un délai moyen de 9,1 ± ET [2-90 jours] jours. Les patients décédés en cours d’hospitalisation avaient consulté dans un délai relativement plus long soit 10,4 jours (p < 0,05). Les causes de décès étaient la septicémie (40%)‚ la méningite (30%)‚ la broncho-pneumopathie (20%) et le paludisme (10%). *Streptococcus pneumoniae* était le germe associé dans 90% des décès.

## Discussion

La fréquence hospitalière des infections retrouvée dans notre étude (21,8%) était proche de celle de Koreissi à Bamako (Mali) et Mabiala à Brazzaville (Congo) qui était respectivement de 26% et de 36,6% [1, 2]. Ces résultats confirment la fréquence des infections chez l'enfant atteint de syndrome drépanocytaire majeur du fait de sa plus grande sensibilité. L’âge moyen de nos patients était proche de celui trouvé par Wierenga en Jamaïque (6,1 ans) [3]. Dans notre étude, les patients drépanocytaires majeurs de 0 à 5 ans étaient la tranche d’âge la plus touchée par les infections dans notre étude alors que Latoundji [4] au Bénin trouve plus d’infections dans la tranche d’âge juste au-dessus (6-10 ans). D’une manière générale, la grande susceptibilité aux infections du sujet drépanocytaire est bien connue [5] et le risque infectieux est maximal chez le jeune patient, en particulier le nourrisson. En plus de l’immaturité physiologique du système immunitaire de l’enfant, une asplénie fonctionnelle (du fait d’infarci spléniques répétés) associée à des anomalies du complément‚ des immunoglobulines‚ de la fonction leucocytaire et de l’immunité à médiation cellulaire pourraient contribuer à fragiliser davantage les moyens de lutte anti-infectieuse du sujet drépanocytaire [3]. Nous avons trouvé 51,9% des patients de sexe féminin porteurs d’infections, ce qui est conforme au résultat d’autres auteurs [3, 4]. Pour l’instant, la prédominance d’un genre ne saurait avoir une explication particulière et serait sans doute le fait du hasard. Notre étude montrait que les infections prédominaient chez les homozygotes SS. Ce résultat est inferieur à celui deà 82,5% pour Gbadoé [6] et 94% pour Latoundji [4] ; la différence pouvant être due à la prise en compte des autres complications aigües chez ces auteurs. Parmi les antécédents, l’épisode infectieux était la circonstance de découverte de 24.8% de nos patients. D’autres auteurs ont retrouvé cette circonstance de découverte dans 13% [7] et 34% [8]. La maladie drépanocytaire ne bénéficiant pas encore d’un programme de lutte spécifique au Burkina Faso, il n’est pas surprenant que la maladie soit découverte dans la grande majorité des cas à l’occasion d’une complication aigue, notamment infectieuse. La couverture vaccinale selon le PEV était bonne chez nos patients (81,2% étaient vaccinés). En Afrique subsaharienne, le taux de couverture vaccinale des enfants drépanocytaires est satisfaisant, allant de 87 à 99% [6, 9, 10]. Les vaccins du PEV sont généralement bien suivis parce qu’ils sont gratuits. En principe, les infections dues au pneumocoque devront se raréfier avec l’introduction en 2013 du vaccin PNEUMO 13 dans le calendrier vaccinal de routine des nourrissons du Burkina Faso. Les vaccins hors du PEV et préconisés par l’OMS pour les drépanocytaires ne sont pas bien administrés. Ils ne sont pas pris en charge dans notre contexte, le système de sécurité sociale étant absent. Ces vaccins sont généralement achetés par les parents des enfants. Nos patients, dans leur majorité, sont allés consulter tardivement, soit parce que la décision était prise tardivement, soit après l’échec d’un traitement dans un centre de santé d’un échelon inférieur.

Dans notre étude, les signes fonctionnels associés à la fièvre étaient par ordre de fréquence décroissante les douleurs ostéoarticulaires‚ la toux et la pâleur. Ils ne diffèrent pas de ceux habituellement décrits dans la littérature [11, 12]. La toux qui prédominait chez les patients de moins de 6 ans pourrait simplement témoigner de l’existence d’un foyer infectieux au niveau respiratoire que l’on sait fréquent à cet âge. Quant aux douleurs ostéo-articulaires avec fièvre, il pourrait s’agir soit de la localisation ostéo-articulaire de l’infection, soit du fait de la fièvre comme facteur déclenchant des crises douloureuses chez le drépanocytaire [11, 12]. Les signes généraux et physiques (pâleur, hépatomégalie, splénomégalie, râles pulmonaires) sont classiquement décrits chez le drépanocytaire [11, 12]. L’hyperleucocytose chez nos patients est classique chez les patients drépanocytaires, même en dehors de tout phénomène infectieux. Cependant, en présence de syndrome infectieux clinique, un taux de leucocytes au-dessus de 20000 leucocytes/mm^3^ est un bon argument en faveur d’une infection [13]. Les examens bactériologiques ont permis d’isoler *Streptococcus pneumoniae* (34,6%) ‚ *Salmonella sp* (32,7%). Le streptocoque et les salmonelles sont plus fréquemment isolés chez les enfants de moins de 6 ans, de même que chez les homozygotes SS. Diop [14] rapportait dans une étude similaire une fréquence de 37% des salmonelles. Wirenga [3] avait dans sa série identifié *Streptococcus pneumoniae* dans 30% et *Haemophilus influenzae* dans 20% des cas. Okuonghae [15] au Nigéria a, quant à lui, trouvé une plus grande fréquence des salmonelles avec 25,9%. Nos résultats sont différents parce que nous avons isolé les germes à partir de plusieurs produits biologiques alors que les auteurs sus cités ont utilisé l’hémoculture seule. Le pneumocoque et les salmonelles sont les deux principaux germes que nous avons retrouvés dans notre étude, accord avec les données de la littérature [13–15]. L’examen parasitologique des selles a permis de retrouver des parasites dans toutes les tranches d’âges et indifféremment chez les patients SS et SC. Les parasites intestinaux mis en évidence étaient exclusivement constitués de protozoaires du type *Giardia intestinalis* (44,4%) et *Entamoeba histolytica* (22,2%). En Afrique sub-saharienne, des auteurs rapportent des fréquences de 38,8% pour la giardiase, 27,8% et 25,9% pour l’amibiase [5, 6]. La différence observée avec notre étude pourrait s’expliquer par la répartition géographique naturelle des parasites. La majorité des drépanocytaires vivent dans les pays en développement où les maladies parasitaires ont une forte prévalence et sévissent de façon endémique. La présence des parasites intestinaux pouvant aggraver leur anémie, il est important de les rechercher régulièrement pour les traiter chez les enfants drépanocytaires.

Les principaux diagnostics retenus chez nos patients étaient représentés par les broncho -pneumopathies (31,6%)‚ le paludisme (16,5%)‚ les ostéomyélites (12,8%) et les septicémies (10,5%). Dembélé [12] avait trouvé que le paludisme venait en tête (28,1%)‚ suivi des ostéomyélites (15,6%) et des pneumopathies (12,5%). Les pneumopathies et les septicémies concernaient plus souvent les patients de 0 à 5 ans ainsi que les formes SS. Nos résultats sont proches de ceux de Koffi [16] en Côte d’Ivoire qui avait retrouvé une prédominance (87%) des infections respiratoires chez les patients SS et chez les patients de moins de 5 ans (59%) [16]. Au Gabon, les pneumopathies viennent également en tête des infections chez le drépanocytaire‚ touchant en majorité les plus jeunes âges [17]. Dans notre étude, le pneumocoque (42,9%) et les salmonelles (28,5%) étaient les principaux pathogènes retrouvés dans les septicémies. Ces bactéries sont retrouvés par d’autres auteurs [3, 15]. Les salmonelles étaient incriminées dans 83,3% des ostéomyélites et 16,7% des cholécystites, ces fréquences étant superposables à celles de Diop [14] qui trouve 90% d’ostéomyélites et 10% de cholécystite dues aux salmonelles. La prépondérance d’Escherichia coli et Klebsiella dans les infections urinaires est conforme aux données de la littérature [18, 19]. Nos patients ont reçu des antibiotiques dans 96,2% des cas, représentés par les céphalosporines de 3^e^ génération avec la ceftriaxone (64,6%)‚ les aminosides avec la gentamicine (48,9%) ‚l'association amoxicilline-acide clavulanique (16,5%). Diagne et al. [8] au Sénégal utilisaient les céphalosporines de 2^ème^ ou 3^ème^ génération dans la prise en charge des infections chez l’enfant drépanocytaire. Pour Gbadoé, au Togo, l’ampicilline, l’amoxicilline-acide clavulanique, les macrolides, les phénicolés, les fluoroquinolones, les céphalosporines et les aminosides sont les antibiotiques de premier choix en fonction du site infectieux, utilisés seuls ou en association [6]. Les antibiotiques utilisés dans notre étude correspondent à ceux de la pratique hospitalière dans notre contexte. Utilisés en association, les antibiotiques ont un effet synergique et sont efficaces contre les germes encapsulés‚ en l’occurrence le pneumocoque et les salmonelles qui sont les germes les plus fréquents dans les infections de l’enfant drépanocytaire. La preuve bactériologique de l’infection n'étant pas toujours apportée, l'antibiothérapie probabiliste reste efficace, devant tout syndrome infectieux chez le sujet drépanocytaire. Le taux de létalité de 7,5% trouvé dans notre étude était plus élevé que celui rapporté par Wierenga [3] qui était de 1,2%. Les décès ont concerné pour 80% les patients homozygotes ; la proportion des homozygotes SS décédés était de 9,5% contre 4% pour les patients SC. En comparaison, Latoundji [4] avait retrouvé un taux de mortalité nul pour les SC et 10,6% pour les SS. Les patients décédés avaient en général consulté après un délai relativement long et présentaient un mauvais état général à l’entrée. Les décès étaient dus pour 40% aux septicémies‚ 30% aux méningites‚ 20% aux pneumopathies et 10% au paludisme.

## Conclusion

Cette étude a permis de montrer que les infections respiratoires et osseuses, les septicémies et le paludisme sont fréquents chez les enfants présentant un syndrome drépanocytaire majeur (SS surtout). Les pathogènes rencontrés sont le streptocoque et les salmonelles. Un diagnostic précoce et un traitement adapté constituent la garantie d’une issue favorable car la létalité liée à ces infections reste élevée.

### Etat des connaissances actuelleS sur le sujet

- 5 à 10% de la population sont drépanocytaires au Burkina Faso;
- Les infections bactériennes sont fréquentes et graves chez les enfants drépanocytaires, en particulier chez ceux porteurs d’une forme majeure.

### Contribution de notre étude à la connaissance

- La fréquence des infections chez le drépanocytaire est élevée, surtout chez les enfants de moins de cinq ans;
- La toux avec fièvre chez l’enfant de moins de 5 ans et la fièvre avec douleurs ostéorticulaires chez l’enfant plus âgé doivent faire suspecter une infection bactérienne chez l’enfant drépanocytaire majeur;
- Le pneumocoque est responsable de septicémie, de méningite et de pneumonie; les salmonelles sont la cause de septicémie, d’ostéomyélite, d’ostéoarthrite et de cholécystite.
